# The Onsager theory of wall-bounded turbulence and Taylor’s momentum anomaly

**DOI:** 10.1098/rsta.2021.0079

**Published:** 2022-03-07

**Authors:** Gregory L. Eyink, Samvit Kumar, Hao Quan

**Affiliations:** ^1^ Department of Applied Mathematics, The Johns Hopkins University, Baltimore, MD 21218, USA; ^2^ Department of Mechanical Engineering, The Johns Hopkins University, Baltimore, MD 21218, USA

**Keywords:** wall turbulence, channel flow, Onsager theory of turbulence, weak solutions, renormalization group, large-eddy simulation

## Abstract

We discuss the Onsager theory of wall-bounded turbulence, analysing the momentum dissipation anomaly hypothesized by Taylor. Turbulent drag laws observed with both smooth and rough walls imply ultraviolet divergences of velocity gradients. These are eliminated by a coarse-graining operation, filtering out small-scale eddies and windowing out near-wall eddies, thus introducing two arbitrary regularization length-scales. The regularized equations for resolved eddies correspond to the weak formulation of the Navier–Stokes equation and contain, in addition to the usual turbulent stress, also an inertial drag force modelling momentum exchange with unresolved near-wall eddies. Using an Onsager-type argument based on the principle of renormalization group invariance, we derive an upper bound on wall friction by a function of Reynolds number determined by the modulus of continuity of the velocity at the wall. Our main result is a deterministic version of Prandtl’s relation between the Blasius −1/4 drag law and the 1/7 power-law profile of the mean streamwise velocity. At higher Reynolds, the von Kármán–Prandtl drag law requires instead a slow logarithmic approach of velocity to zero at the wall. We discuss briefly also the large-eddy simulation of wall-bounded flows and use of iterative renormalization group methods to establish universal statistics in the inertial sublayer.

This article is part of the theme issue ‘Scaling the turbulence edifice (part 1)’.

## 1. Introduction

A recent paper of Bardos & Titi [1] has initiated the mathematical investigation of Onsager’s ‘ideal turbulence’ theory for wall-bounded flows, already followed by several works with improvements [2–4]. These papers have all pursued the line of Onsager’s original mathematical analysis^1^ from around 1945 [5–7], the details of which he never published but which were recovered and successively improved in several works of others about 50 years later [10–13]. The object of all of these works was to identify suitable conditions for conservation of kinetic energy by Euler solutions or, more physically stated, the conditions that must be violated in order for energy dissipation to remain non-zero in the limit of infinite Reynolds number for Navier–Stokes solutions. For wall-bounded flows such as pipe and channel flow or developing boundary layers over a flat plate, we believe, however, that it is even more straightfoward and illuminating to consider dissipative anomalies in the conservation of *linear momentum*. It is interesting that both types of anomalies were first suggested by G. I. Taylor, for kinetic energy dissipation in a 1917 report [14] and for momentum dissipation even earlier in a 1915 paper in the present journal on eddy motion in the atmosphere [15]. We quote Taylor from the final pages of his paper:

…a very large amount of momentum is communicated by means of eddies from the atmosphere to the ground. This momentum must ultimately pass from the eddies to the ground by means of the almost infinitesimal viscosity of the air. The actual value of the viscosity of the air does not affect the rate at which momentum is communicated to the ground, although it is the agent by means of which the transference is effected …

The finite loss of momentum at the walls due to an infinitesimal viscosity may be compared with the finite loss of energy due to infinitesimal viscosity at a surface of discontinuity in a gas.${\;}^{*}$ [15]

The article referenced by Taylor with the asterisk ‘*’ in this quotation is his own 1910 paper on shock discontinuities [16]. It is remarkable that Taylor in this early paper not only recognized that there could be a finite loss of momentum due to an ‘infinitesimal viscosity’ but also compared this phenomenon with discontinuous shock solutions which we now understand to be described, in modern language, by weak solutions of inviscid fluid equations.

Walls or solid boundaries are certainly required for anomalous dissipation of momentum by weak solutions of Euler or Navier–Stokes, because these equations in the flow interior express nothing other than local conservation of the linear momentum, in the form

1.1 $$\frac{\partial \text{u}}{\partial t}+\nabla \cdot \text{T}=0,$$

with the stress tensor $\rho T_{ij}$ representing flux in the ith coordinate direction of the conserved jth component of momentum $\rho u_{j}$, for mass density ρ and fluid velocity u. This stress tensor for incompressible Navier–Stokes may be taken in the form

1.2 T=uu+pI−ν∇u,

with p=P/ρ the kinematic pressure and ν=η/ρ the kinematic viscosity. The stress tensor is the ultimate vehicle of transmission of momentum to the wall, via its normal component

1.3 $$\text{n}\cdot \text{T}=p\text{n}-\frac{\nu \partial \text{u}}{\partial n},$$

which produces both viscous drag due to the Newtonian wall stress and form drag due to the pressure distribution, acting as sinks of momentum.

In addition, however, a spatial cascade is required which transfers momentum via ideal nonlinear interactions through an ‘inertial sublayer’ in order to maintain the necessary supply from the flow interior, in close analogy with the cascade of kinetic energy through the ‘inertial subrange’ in fluid turbulence away from walls [17]. The aim of the present short paper is to discuss this momentum cascade in wall-bounded turbulence using the same methods which have been previously applied to analyse anomalous dissipation of kinetic energy in such wall-bounded flows [1–4]. To be concrete, we shall consider the specific example of pressure-driven flows through pipes and channels. In this brief summary, we shall focus mainly on the essential ideas and on the physical motivation of the mathematical approach, with full details and rigorous analysis provided in a longer paper [18]. The conclusion of the analysis is that certain specific types of discontinuity of the velocity at the wall are required for a strict momentum anomaly, in agreement with the early insight of Taylor [15] and with similar conclusions for kinetic energy anomaly [1–4]. In fact, we shall see that even a ‘weak anomaly’ in momentum conservation requires a less than smooth approach of the streamwise velocity to zero as the distance to the wall is decreased through the inertial sublayer, in analogy also to energy dissipation [19,20]. We obtain in this manner a version of Prandtl’s relation between the Blasius drag law $f(Re)∼{Re}^{-1/4}$ and a (1/7)th power-law approach of the streamwise velocity to zero ([21,22], and see also [23], pp. 599–600), but now rigorously valid for individual flow realizations without ensemble averaging. At asymptotically large Reynolds numbers, we can similarly show that the von Kármán–Prandtl drag law requires a logarithmically slow approach of the streamwise velocity to zero at the wall [18].

The theoretical analysis of Onsager [5–7] is best understood physically as an application of the ‘principle of renormalization group invariance’ [24], as we have previously explained for turbulence away from walls [25]. This same interpretation holds also for wall-bounded turbulence, although the mode-elimination must now involve not only filtering out small-scale eddies but also windowing out near-wall eddies. This form of coarse-graining produces the usual turbulent ‘subgrid’ stress but in addition an ‘inertial drag force’ which models the influence of the ignored near-wall eddies. This elimination scheme therefore offers hope for a new systematic approach to large-eddy simulation (LES) of wall-bounded turbulent flows, which we shall briefly discuss. An iterative application of such a renormalization group scheme seems natural to investigate conjectured universality in wall-bounded turbulence, such as Townsend’s similarity hypothesis on rough walls [26], with elimination and rescaling performed in successive steps as in Wilsonian renormalization group (RG). We discuss briefly also these wider possibilities.

## 2. Formulation of the problem

We begin with a concise summary of the empirical evidence on the Reynolds-scaling of the friction factor in turbulent pipes and channels, obtained from both laboratory experiments and numerical simulations. First, however, an important distinction must be made between walls which are ‘smooth’ or ‘rough’ in the hydraulic sense and in the mathematical sense, since the meanings are quite different. No physical wall can ever be specularly smooth but must always have some slight irregularities or corrugations with height k and the wall is considered ‘hydraulically rough’ if the dimensionless height (roughness Reynolds number) $k_{+}=u_{\tau }k/\nu$ is at least order unity. Here, $u_{\tau }$ is the ‘friction velocity’, which measures the flux of momentum through the ‘inertial sublayer’ [17]. It is defined in the channel by $\rho u_{\tau }^{2}=\gamma H$, where $\gamma =-\partial \bar{p}/\partial x$ is the mean streamwise pressure-gradient and H is the channel half-width, and in the circular pipe by $2\rho u_{\tau }^{2}=\gamma H$ with H the pipe radius. Note that a ‘hydraulically rough’ wall can be either smooth or rough/singular in the mathematical sense, since a wall surface would be considered ‘hydraulically rough’ if its height profile were given either by a smooth sinusoidal profile h(x)=ksin⁡x or by a non-differentiable sawtooth function h(x)=2k|x−⌊x⌉| (where ⌊x⌉ is the nearest integer to x) if $k_{+}≳1$. When we refer below to ‘smooth’ or to ‘rough’ walls, we shall always use these terms with the hydrodynamic interpretation and not in the mathematical sense of differentiable manifold versus more singular surface.

The friction factor may be defined by $f(Re,k/H):=\gamma H/\frac{1}{2}\rho {\bar{U}}^{2}$, where $\bar{U}$ is the mean streamwise velocity averaged over the cross-sectional area of the pipe or channel, and its limit at high Reynolds numbers $Re=\bar{U}(2H)/\nu \gg 1$ depends very crucially on the wall roughness. It is only for rough walls that there is a strict ‘dissipative anomaly’, with f(Re,k/H) tending to a constant value $f_{*}(k/H)$ as Re→∞, as indicated both by experiments [27–29] and simulations [30]. By contrast, smooth-wall pipe flow may be idealized by taking k=0 and it is observed that f(Re)→0 as Re→∞ [31–36]. Roughly, the laminar value f∼32/Re is observed for $Re≲10^{3}$, followed by the Blasius law $f∼c\;{Re}^{-1/4}$ for $10^{3}≲Re≲10^{5}$, and then the von Kármán–Prandtl Law [37,38] given implicitly by $1/\sqrt{f}=a\log(Re\sqrt{f})+b$ for $Re≳10^{5}$, with suitable constants a, b, c. In fact, the above summary only roughly states the most common interpretation of the observations and there are many refinements and alternative proposals. In particular, there is an old idea of Prandtl and others that the drag may be power-law $f(Re)∼{Re}^{-p}$ with an exponent p(Re) that tends slowly to 0 as Re→∞; for example, see [23], Ch XXa for the classical literature and [39,40] for a modern reincarnation. Despite intense ongoing discussion of the details, the observations support unequivocally the fact that f(Re) decays much slower than the laminar rate 1/Re as Re→∞. Following the terminology introduced for kinetic energy dissipation [19,20], we may refer to this sublaminar decay as a ‘weak dissipative anomaly’, in contrast to the strong dissipative anomaly with a non-vanishing limit that is observed for rough walls. We discuss here further the case of a finite (but large) range of Re in the Blasius range.

The dynamical consequences of these anomalies can be most easily understood by an examination of the global momentum balance. We consider as the simplest situation a channel flow in domain Ω which is periodic both in the streamwise direction x∈[−L,L] and in the spanwise direction z∈[−W,W] and with the vertical coordinate y constrained to lie between two surfaces $y=H_{-}(x,z)$ and $y=H_{+}(x,z)$, where $H_{\pm }(x,z)$ are smooth functions that satisfy the conditions $|H_{\pm }(x,z)\mp H|\leq k$ to model hydraulically rough walls. The governing equation is incompressible Navier–Stokes

2.1 $$\frac{\partial \text{u}}{\partial t}+\nabla \cdot [\text{uu}+p_{T}\text{I}-\nu \nabla \text{u}]=0,\;\nabla \cdot \text{u}=0,$$

where the total pressure is given by $p_{T}=p-\gamma x$ with p(x,t) periodic in x and z and with γ an applied pressure gradient constant in space and time. The corresponding global momentum balance is easily derived formally^2^ as

2.2 $$\int_{\Omega }\text{u}(t)\;\text{d}V-\int_{\Omega }\text{u}(0)\;\text{d}V=\int_{0}^{t}\;\text{d}s\int_{\partial \Omega }\left(p_{T}\text{n}-\nu \frac{\partial \text{u}}{\partial n}\right)\;\text{d}A+\gamma |\Omega |t\hat{\text{x}},$$

where the boundary $\partial \Omega =\partial \Omega_{+}\cup \partial \Omega_{-}$ with $\partial \Omega_{\pm }=\{(x,H_{\pm }(x,z),z):\;x\in [-L,L],z\in [-W,W]\}$, n is the unit normal vector at the boundary pointing inward to the fluid, |Ω| is the volume of the domain and $\hat{x}$ is a unit vector in the streamwise direction. Denoting the velocity components as u=(u,v,w), the quantity appearing in the streamwise momentum balance

2.3 $$\tau_{w}:=\rho \frac{H}{|\Omega |}\int_{\partial \Omega }\left(-p_{T}n_{x}+\nu \frac{\partial u}{\partial n}\right)\;\text{d}A,$$

represents the instantaneous wall stress, whose long-time average in the steady-state equals γH and thus counters the applied pressure head.

Note that for k≪H, |Ω|≃A(2H) with A≃(2W)(2L) the area of a single face of the wall and thus $\tau_{W}$ is an area average of two contributions, the ‘form drag’ from pressure asymmetry on the roughness elements and the ‘viscous drag’ from the Newtonian stress. The friction factor may then be written in terms of its long-time average as $f(Re,k/H)={\bar{\tau }}_{w}/\frac{1}{2}\rho {\bar{U}}^{2}$. For smooth walls with k=0, the wall drag is purely viscous and the ‘weak anomaly’ which is observed empirically implies that the vertical derivatives of streamwise velocity, ∂u/∂y, must diverge at the wall as Re→∞, for otherwise f(Re)=O(1/Re) if those derivatives remain smooth. This is in addition to any divergences of velocity-gradients in the flow interior that are required by the slowly vanishing energy dissipation there [20]. In the case of rough walls, the viscous drag is observed to vanish slowly, just as for smooth walls, so that the asymptotic drag $f_{*}(k/H)$ is due entirely to the form drag on the roughness elements in the limit [30]. The non-vanishing drag with rough walls thus has the same origin as the non-vanishing drag for flow past finite solid bodies and analogous flow phenomena are observed near the roughness elements, such as separating boundary layers and pressure asymmetry [30,42,43]. As recently shown [44] (also [45]), the associated form drag must in fact be due to a flux of spanwise vorticity across the streamwise flow. Although the viscous drag becomes negligible in the limit, it is again observed to vanish more slowly than 1/Re so that the wall-normal derivatives ∂u/∂n must diverge as Re→∞.

These divergences of velocity-gradients at the wall, as well as the additional divergences in the interior, can be described as an ‘ultraviolet catastrophe’. They imply that a naive interpretation of the fluid equations (2.1) as partial differential equations is no longer possible when Re→∞. As in quantum field theory and critical phenomena, the development of a valid dynamical description in that limit requires a *regularization* of these divergences [24]. Regularizing by a suitable ‘coarse-graining’ operation, the resulting regularized dynamics in fact corresponds to what in mathematics is called a *weak formulation* of the fluid equations: see [46], §2 or [25]. In this formulation, one may pass to the limit Re→∞ and, under physically reasonable assumptions consistent with observations, the limits (along suitable subsequences) exist and are described as *weak Euler solutions* [47], in agreement with the ideas of Taylor and Onsager. In the following section, we explain in physical terms the regularization that has been employed in the recent mathematical literature on wall-bounded flows [1–4] and explore its consequences.

## 3. Onsager RG approach and inertial drag force

To regularize divergences of gradients in the interior, a spatial coarse-graining/low-pass filtering/mollifying operator may be applied to the velocity field by convolving it with a smooth filter kernel $G_{\ell }(r)=\ell^{-3}G(r/\ell )$, denoted ${\bar{u}}_{\ell }=G_{\ell }*u$, which corresponds to ignoring eddies of size <ℓ [11,25,48]. It is convenient to assume that the filter kernel G is supported in a ball of radius 1. It then follows that the definition of ${\bar{u}}_{\ell }(x,t)$ makes sense for points x∈Ω with distance at least ℓ from the boundary ∂Ω. To eliminate also the divergences of the velocity-gradients at the wall and to obtain, a well-defined coarse-grained velocity, one must also smoothly ‘window out’ eddies at distances <h to the wall, with h>ℓ. This is accomplished by taking a smooth windowing function $\eta_{h,\ell }(\delta )$ with the properties that

3.1 $$\eta_{h,\ell }(\delta )=\left\{ \begin{array}{ll} 0 & \delta <h\\ 1 & \delta >h+\ell \end{array}\right.,$$

and $\eta_{h,\ell }(\delta )$ monotone increasing on the interval [h,h+ℓ]. See [2], footnote 4 for a mathematical recipe to construct such a function and note that its kth-derivative can be estimated as $||\eta_{h,\ell }^{\left(k\right)}|{|}_{\infty }=O(\ell^{-k})$. Finally, one defines for all x∈Ω

3.2 $${\tilde{\text{u}}}_{\ell ,h}(\text{x},t)=\eta_{\ell ,h}(d(\text{x})){\bar{\text{u}}}_{\ell }(\text{x},t),$$

where d(x) is a suitable ‘distance function’ which measures the distance of x∈Ω to the boundary. There is considerable freedom in the choice of this function. In the current mathematics literature [1–4], the function $d:\Omega \to R^{+}$ has been defined by

3.3 $$d(\text{x})=\underset{\text{y}\in \partial \Omega }{\inf}|\text{x}-\text{y}|.$$

With this definition $\nabla d(x)=n(y_{x}):=n(x)$, where n(y) is the inward-pointing unit normal vector at a point y∈∂Ω and $y_{x}\in \partial \Omega$ is the point at which the infimum in (3.3) is achieved for each x∈Ω. See [1]. However, other choices of distance function might be more useful for some purposes, e.g. d(x)=min{|y−H|,|y+H|} could be useful in an iterative RG analysis of a rough-wall channel flow in order to establish universal statistics in the ‘inertial sublayer’. A similar type of ‘time windowing’ was employed in the recent RG analysis of Lagrangian spontaneous stochasticity [49], where it corresponds to ignoring non-universal initial times of the particle position histories. The coarse-grained velocity defined by (3.2) may be described picturesquely as the fluid velocity seen by an observer who is myopic and who also has tunnel vision, with parameter ℓ characterizing the blurriness of their eyesight and h their loss of peripheral vision.

With this definition, it is then straightforward to derive from (2.1) the following regularized dynamical equation:

3.4 $$\frac{\partial {\tilde{\text{u}}}_{\ell ,h}}{\partial t}+\nabla \cdot [{\tilde{\tau }}_{\ell ,h}(\text{u},\text{u})+{\tilde{\text{u}}}_{\ell ,h}{\tilde{\text{u}}}_{\ell ,h}+{\tilde{p}}_{\ell ,h}\text{I}-\nu {\tilde{\nabla \text{u}}}_{\ell ,h}]={\text{f}}_{\ell ,h}+{\tilde{\gamma }}_{\ell ,h}\hat{\text{x}},$$

where the *turbulent (or subgrid) stress* may be defined as usual by

3.5 $${\tilde{\tau }}_{\ell ,h}(\text{u},\text{u})={\left(\tilde{\text{uu}}\right)}_{\ell ,h}-{\tilde{\text{u}}}_{\ell ,h}{\tilde{\text{u}}}_{\ell ,h}$$

and, in addition, a new *inertial drag force* appears associated with the eliminated near-wall eddies

3.6 $${\text{f}}_{\ell ,h}=\nabla \eta_{\ell ,h}\cdot {\bar{\text{T}}}_{\ell }=\eta_{\ell ,h}^{\prime }(d(\text{x}))\;\text{n}(\text{x})\cdot [{\left(\bar{\text{uu}}\right)}_{\ell }+{\bar{p}}_{\ell }\text{I}-\nu \nabla {\bar{\text{u}}}_{\ell }],$$

and which represents momentum-exchange with those unresolved eddies. Note that this force is dominated by inertial dynamics only for Re≫1 at fixed ℓ, h, when the final viscous contribution becomes negligible, but that latter term must be retained if ℓ, h are permitted to be Re-dependent, as we allow below. Mathematically, the regularized equation (3.4) when considered for all possible choices of h>ℓ is equivalent to the standard weak formulation of the incompressible Navier–Stokes equation (see [41], Ch. V.1.2).

One important potential application of the regularized equations (3.4) is to provide the basis for an LES of wall-bounded flows. The proper modelling of walls and solid boundaries is currently considered one of the most pressing problems in making LES a practical engineering tool [50]. In this context, both the subgrid stress ${\tilde{\tau }}_{\ell ,h}$ and the inertial drag force $f_{\ell ,h}$ must be modelled. If length-scale ℓ is chosen in the inertial sub-range and distance h is chosen in the inertial sublayer, then one can expect that these quantities have universal statistical properties independent of the small-scale dissipation and of the detailed properties of the wall. In addition, however, another quantity must be modelled which appears in the coarse-grained mass balance

3.7 $$\nabla \cdot {\tilde{\text{u}}}_{\ell ,h}={\tilde{\sigma }}_{\ell ,h},$$

which we call the *inertial mass source*

3.8 $${\tilde{\sigma }}_{\ell ,h}:=\eta_{h,\ell }^{\prime }(d(\text{x}))\text{n}(\text{x})\cdot {\bar{\text{u}}}_{\ell }.$$

This quantity measures the mass-exchange with the unresolved near-wall eddies and it is non-vanishing only for h<d(x)<h+ℓ. The windowing operation has thus introduced effective ‘compressibility’, which causes some slight complications for mathematical analysis and for numerical solution. In particular, the Poisson equation for coarse-grained pressure becomes

3.9 $$-△{\tilde{p}}_{\ell ,h}=\partial_{t}{\tilde{\sigma }}_{\ell ,h}+\nabla \nabla :[{\tilde{\text{u}}}_{\ell ,h}{\tilde{\text{u}}}_{\ell ,h}+{\tilde{\tau }}_{\ell ,h}-\nu {\tilde{\nabla \text{u}}}_{\ell ,h}]-\nabla \cdot {\text{f}}_{h,\ell }$$

which involves derivatives of all three modelled quantities but which can yield the resolved pressure by applying standard Poisson solvers.

The regularized equation (3.4) involves two arbitrary lengths ℓ and h and, as well, three arbitrary functions G, η and d. The ‘principle of renormalization group invariance’ is that no objective physics can depend upon these arbitrary quantities introduced for the purpose of regularization [24,25]. The present example is a case of a several-parameter renormalization group involving changes of the entire regularization scheme, which was encountered already in quantum field theory [51–53] and which has been applied since to PDE’s, including boundary-value problems [54,55]. A key idea in RG methods is that the arbitrariness in the regularization parameters may be exploited by choosing them in some optimal way to deduce non-trivial consequences. We shall describe one application of that principle in the following section.

## 4. Continuity at the wall and bounds on the friction factor

The core of Onsager’s original argument for a one-third Hölder singularity of ideal Euler solutions was a rigorous upper bound on energy flux in terms of velocity increments [5–7], which was developed and improved in subsequent works [10–13]. An analogous result was obtained also for Navier–Stokes solutions at finite Re in [20], where a power-law bound on viscous energy dissipation of the form $\varepsilon /(u_{\text{rms}}^{3}/L)\leq C{Re}^{-(3h-1)/(1+h)}$ was obtained from the assumption of Hölder-type regularity with exponent h uniform in the Reynolds number Re. It follows from this bound that even a ‘weak dissipative anomaly’ requires quasi-singularities, or loss of uniform regularity of the Navier–Stokes solutions. The estimate in [20] was obtained by an RG-type argument, considering the balance equation for unresolved kinetic energy $k_{\ell }=(1/2)\text{tr}(\tau_{\ell })$ and then optimizing the bound with respect to the arbitrary regularization scale ℓ. This optimization required a balance of the energy flux and the resolved viscous dissipation, which selected an optimal length scale $\ell_{*}∼L{Re}^{-1/(1+h)}$ that coincides with the Kolmogorov length for h=1/3.

We wish to obtain a similar bound on the friction factor f(Re) in turbulent channel flow by an analogous argument based on the momentum balance equation of the unresolved eddies, associated with their velocity field

4.1 $${\text{u}}_{\ell ,h}^{\prime }:=\text{u}-{\tilde{\text{u}}}_{l,h}.$$

The corresponding momentum balance is easily obtained by subtracting (2.1) and (3.4)

4.2 $$\frac{\partial {\text{u}}_{\ell ,h}^{\prime }}{\partial t}+\nabla \cdot [{\left(\text{uu}\right)}_{h,\ell }^{\prime }+{\left(p_{T}\text{I}-\nu \nabla \text{u}\right)}_{h,\ell }^{\prime }]=-{\text{f}}_{h,\ell }+(1-\eta_{h,\ell })\gamma \hat{\text{x}}.$$

Here, we have defined in general $A_{\ell ,h}^{\prime }:=A-{\tilde{A}}_{l,h}$ and we note that, on the boundary ∂Ω, $A_{\ell ,h}^{\prime }=A$. Specializing to the *smooth wall* case for simplicity, we then obtain the global balance of streamwise momentum of the unresolved eddies by integrating over the space–time domain

4.3 $$\begin{array}{rl} \frac{1}{\rho }\tau_{w}^{\left(T\right)} & \;:=\frac{1}{T}\int_{0}^{T}\;\text{d}t\frac{1}{|\partial \Omega |}\int_{\partial \Omega }\nu \frac{\partial u}{\partial n}\;\text{d}A=-\frac{1}{T}\int_{0}^{T}\;\text{d}t\frac{1}{|\partial \Omega |}\int_{\Omega_{h+\ell }\setminus \Omega_{h}}f_{h,\ell }^{x}\;\text{d}V\\ & \;\;-\frac{1}{T}\left[\frac{1}{|\partial \Omega |}\int_{\Omega }(u_{h,\ell }^{\prime }(T)-u_{h,\ell }^{\prime }(0))\;\text{d}V\right]+\gamma \frac{1}{|\partial \Omega |}\int_{\Omega_{h+\ell }}(1-\eta_{h,\ell })\;\text{d}V. \end{array}$$

Here, we have defined $\Omega_{h}=\{(x,y,z)\in \Omega :\;y>H-h\text{ or }y<h-H\}$ which is the set of points within distance h of ∂Ω and we have noted that the inertial drag force $f_{h,\ell }$ is non-zero only in the layer $\Omega_{h+\ell }\setminus \Omega_{h}$ of thickness ℓ.

The dominant balance in (4.3) is between the two terms in the first line, when h,ℓ≪H. In fact, the final term from the applied pressure gradient is of order O(γ(h+ℓ)) and the other term in the second line from the time-derivative is $O(\bar{U}H/T{\left(\ell /H\right)}^{\sigma })$ with σ≐1/3 when ℓ is chosen in the inertial range at wall distance h and even smaller at those h,ℓ where the velocity field is smooth. The main term in (4.3) from the inertial drag force has two contributions that follow from

4.4 $$f_{\ell ,h}^{x}=\eta_{\ell ,h}^{\prime }(y)[{\bar{\left(uv\right)}}_{\ell }-\nu \frac{\partial {\bar{u}}_{\ell }}{\partial y}].$$

The magnitude of these two contributions depends upon the scaling properties of two flow quantities ${\left\langle u\right\rangle }_{h,\ell ,T},{\left\langle uv\right\rangle }_{h,\ell ,T}$ defined by the following averaging operation:

4.5 $${\left\langle A\right\rangle }_{h,\ell ,T}:=\frac{1}{|\Omega_{h+2\ell }\setminus \Omega_{h-\ell }|}\int_{\left(\Omega_{h+2\ell }\setminus \Omega_{h-\ell }\right)}\left|\frac{1}{T}\int_{0}^{T}A\;\text{d}t\right|\;\text{d}V$$

which represents a space $L^{1}(\Omega_{h+2\ell }\setminus \Omega_{h-\ell })$-norm of the time-average over interval (0,T). Note that the slab $\left(\Omega_{h+\ell }\setminus \Omega_{h}\right)$ which supports the inertial drag force has been thickened by ℓ, because the quantities that appear in (4.4) are smeared over that length-scale. Here, ${\left\langle u\right\rangle }_{h,\ell ,T},{\left\langle uv\right\rangle }_{h,\ell ,T}$ appear like typical quantities in the statistical theory of wall-bounded turbulence, a mean streamwise velocity and a Reynolds stress. However, it should be kept in mind that the limit T→∞ is not required but only sufficiently large T is needed so that term $O(\bar{U}H/T{\left(\ell /H\right)}^{\sigma })$ may be neglected, and these averages are thus properties of an individual solution, not an ensemble.

To illustrate the scaling hypotheses that we employ, we consider first the intermediate Reynolds numbers $10^{3}≲Re≲10^{5}$ where the Blasius drag law is observed. We assume that with n,m∈(0,1/3)

4.6 $${\left\langle u\right\rangle }_{h,\ell ,T}\leq A\bar{U}(\frac{h}{H}{)}^{m}$$

and

4.7 $${\left\langle uv\right\rangle }_{h,\ell ,T}\leq B{\bar{U}}^{2}(\frac{h}{H}{)}^{m+n}.$$

These inequalities express the possible rate of approach of the streamwise velocity u and the velocity product uv to their value 0 at the boundary, so that m, n may be interpreted as Hölder-type exponents of the boundary continuity. Since ${\left\langle A\right\rangle }_{h,\ell ,T}\leq ||A|{|}_{L^{1}((\Omega_{h+\ell }\setminus \Omega_{h})\times (0,T))}$, we could substitute the latter $L^{1}$-norm in the above hypotheses, but we expect that bounds with the averaging operation are sharper. As a matter of fact, the relations (4.6)and (4.7) are expected to hold at least for large T as near equalities with m≃n≃1/7, which we shall verify with numerical channel-flow data below. The power-law profile for the mean velocity $\langle u(y)\rangle ∼\bar{U}{\left(y/H\right)}^{1/7}$ has been known since the study of Nikuradse [31] to hold for pipe flow over nearly the entire radius, in the Reynolds range $10^{3}≲Re≲10^{5}$ (with a possible slow decrease of m with Re) and, in fact, we require the bounds (4.6) and (4.7) only for averages in the slab $\Omega_{h+2l}\setminus \Omega_{h-l}$ and not over the entire height of the channel.

Using (4.3), (4.4), (4.6), (4.7), it is relatively straightforward to obtain an estimate on the friction factor averaged over times 0<t<T, of the form

4.8 $$f^{\left(T\right)}:=\frac{\tau_{w}^{\left(T\right)}}{(1/2)\rho {\bar{U}}^{2}}\leq A^{\prime }\frac{\nu }{\bar{U}\ell }{\left(\frac{h}{H}\right)}^{m}+B^{\prime }{\left(\frac{h}{H}\right)}^{m+n},$$

with appropriate constants $A^{\prime }$, $B^{\prime }$ that depend upon A, B in (4.6) and (4.7) and functions G, η and d. For full details, see [18]. Care must be taken to optimize these constants to obtain the tightest bound, since the scaling assumptions are valid over only a finite range of Re. Further optimizing with respect to the length scales h and ℓ yields optimal values $\ell_{*}=h_{*}$ and

4.9 $$h_{*}=c_{*}H\;{Re}^{-1/(1+n)},$$

for some constant $c_{*}$, yielding our final upper bound

4.10 $$f^{\left(T\right)}\leq C^{\prime }\;{Re}^{-(m+n)/(1+n)}.$$

If $f^{\left(T\right)}≃C\;{Re}^{-p}$ over the range $10^{3}≲Re≲10^{5}$ and if the constant $C^{\prime }$ is not much greater than C, then we can rigorously infer from (4.10) that (m+n)/(1+n)≤p. In particular, if m=n, then n≤p/(2−p), which gives n≤1/7 for p=1/4. If, in fact, m=n and p=2n/(1+n), then since $f\propto {\left(u_{\tau }/\bar{U}\right)}^{2}$, it follows that ${Re}_{\tau }:=u_{\tau }H/\nu =C\;{Re}^{1/(1+n)}$ and thus (4.9) is equivalent to

4.11 $$h_{*}=\frac{c_{*}^{\prime }\nu }{u_{\tau }},$$

for some constant $c_{*}^{\prime }$, or $h_{*}^{+}=c_{*}^{\prime }$ in standard ‘wall units’ (denoted by + superscript) where all quantities are non-dimensionalized with ν and $u_{\tau }$. This should not be surprising, since our mathematical optimization corresponds to balancing the contributions from the Reynolds stress and the viscous stress in the inertial drag force (4.4) and this is the standard definition of the ‘buffer layer’, which occurs conventionally around $h^{+}∼12$ [17].

**Figure 1.**
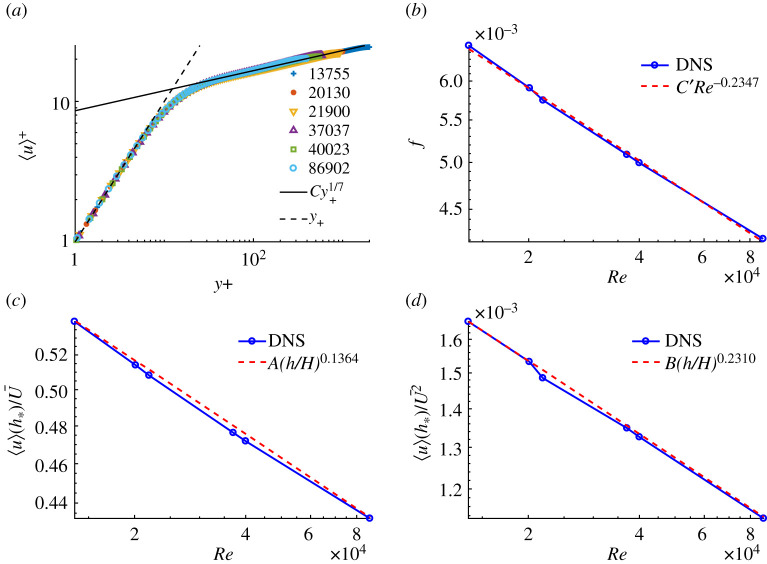
Channel-flow DNS data of Moser *et al.* [56], Del Alamo *et al.* [57], Hoyas & Jiménez [58] and the JHTDB database [59]. (*a*) Mean streamwise velocity profile, in wall units; (*b*) friction factor f(Re) versus Re; (*c*) Mean streamwise velocity and (*d*) mean Reynolds stress at $h_{+}^{*}=12$ versus Re. The red dashed lines in (*b*–*d*) are power-law fits. (Online version in colour.)

To illustrate that our hypothesized bounds (4.6) and (4.7) are realistic, even as near equalities, we show data taken from a compilation of several numerical channel flow simulations [56–59], to cover a range of Reynolds numbers $Re=2H\bar{U}/\nu =13755\text{--}86902$ $\left({Re}_{\tau }=395-2000\right)$. See figure 1. These mean data all correspond to the limit T→∞ in our analysis. We see in figure 1*a* that the 1/7 power law is a good fit to the mean streamwise velocity profile over most of the channel width, except in the viscous sublayer where $u_{+}∼y_{+}$. Best fits reveal, in fact, that the power m is slowly decreasing with Re. Figure 1*b* shows that the drag law is Blasius-like, but with an exponent somewhat smaller than 1/4. The plots in figure 1*c,d* show that our hypotheses (4.6) and (4.7) are reasonable if one takes $h=12\nu /u_{\tau }$, with both mean streamwise velocity and Reynolds stress exhibiting near power laws. From the best-fit exponents, we find that (m+n)/(1+n)≐0.211 which may be compared with p≐0.2347. The agreement is very reasonable given that the simulations considered use different codes and numerical resolutions and have somewhat different aspect ratios. It would obviously be better to have data from several runs of a single numerical scheme over a span of Reynolds numbers.

Because our results are closely related to those of Prandtl [21–23], we must briefly compare both our methods and our conclusions. In what is now standard practice [33], Prandtl very simply obtained the drag law by averaging the mean streamwise velocity profile in the form

4.12 $$\langle u(y)\rangle =Au_{\tau }y_{+}^{m},\;y_{+}=\frac{u_{\tau }y}{\nu },$$

from $y_{+}=0$ to $y_{+}=H_{+}={Re}_{\tau }$, to obtain the relation $\bar{U}/u_{\tau }=A\;{Re}_{\tau }^{m}/(m+1)$. As a consequence,

4.13 $$\langle u(y)\rangle =(m+1)\bar{U}{\left(\frac{y}{H}\right)}^{m},$$

which is a version of our first scaling hypothesis (4.6). Furthermore, $Re:=\bar{U}(2H)/\nu =2A\;{Re}_{\tau }^{m+1}/(m+1)$ and this yields the drag law

4.14 $$f:=2{\left(\frac{u_{\tau }}{\bar{U}}\right)}^{2}=C\;{Re}^{-2m/(1+m)},\;C=8{\left(\frac{m+1}{2A}\right)}^{2/(m+1)}.$$

No explicit use is made of momentum balance in Prandtl’s argument. However, the mean momentum balance in its standard form [17]

4.15 $$-\langle u^{\prime }v^{\prime }\rangle +\frac{\nu \partial \langle u\rangle }{\partial y}=u_{\tau }^{2}\left(1-\frac{y}{H}\right)≐u_{\tau }^{2},\;y\ll H$$

implies that in the buffer layer with $y_{*}≐12\nu /u_{\tau }$

4.16 $$-\langle u^{\prime }v^{\prime }\rangle ≐\left(\frac{1}{2}\right)u_{\tau }^{2}\propto {\bar{U}}^{2}\;{Re}_{\tau }^{-2m}\propto {\bar{U}}^{2}{\left(\frac{y_{*}}{H}\right)}^{2m}$$

which is a version of our second scaling hypothesis (4.7) with n=m.

The difference between our result (4.10) and the traditional one of Prandtl (4.14) is that our result is valid (as an inequality) for individual flow realizations, without taking a limit T→∞. In addition, our scaling hypotheses (4.6) and (4.7) need hold only as inequalities and only over the thin slab $\Omega_{h+2\ell }\setminus \Omega_{h-\ell }$ near the wall, whereas Prandtl required strict power-law profiles over nearly the entire extent of the pipe or channel. It is interesting, incidentally, that recent precision measurements on pipe flow [60] show that the Blasius −1/4 law holds with quite high accuracy up to $Re≐7\times 10^{4}$ and then, in agreement with earlier studies [61], some sort of structural transition occurs at this Reynolds number. It is worth checking whether the 1/7th power-law profile holds also in pipe-flow to high precision for the intermediate range of Reynolds numbers. An alternative explanation exists for the Blasius law in terms of the ‘spectral link’ which connects it with the scaling of the Kolmogorov dissipation velocity [62–64] but it is not yet clear how to derive such a result from our analysis. At asymptotically high Reynolds numbers, the von Kármán–Prandtl logarithmic drag law appears to be well-satisfied [32–36]. Our arguments again apply and now imply that a logarithmically slow approach of streamwise velocity to 0 at the wall is required [18].

## 5. Conclusion

In this paper, we have outlined the application of Onsager’s RG-type arguments to turbulent pipe and channel flow, focusing mainly on the case of hydraulically smooth walls. As a first concrete result, we have derived a deterministic version of Prandtl’s relation between power-law scaling of wall friction and power-law profiles of mean streamwise velocity, but now interpreted in terms of continuity properties of velocity fields at the wall.

The results concerning power laws in this short survey apply physically only to an intermediate range $10^{3}≲Re≲10^{5}$, but it is worth speculating briefly about the limit Re→∞. Based upon the von Kármán–Prandtl theory [37,38], one can expect that the limiting Euler solution for the case of a smooth wall is simple plug flow, with a uniform velocity profile and zero wall friction. This is also the recent conclusion of Cantwell [65] who argues that the asymptotic velocity must be ‘plug flow with a vanishingly thin viscous wall layer’, in agreement with the discontinuity at the wall suggested by Taylor [15]. To obtain a less trivial limiting Euler solution with a strict dissipative anomaly (non-vanishing drag) one must consider pipe and channel flow with hydraulically rough walls or, alternatively, flow past a finite solid body. These two classes of flows are very similar, with limiting dissipative anomaly due to form drag from asymmetric pressure distributions and associated spanwise vorticity flux across the streamwise flow [44].

The coarse-graining employed here, with two length-scales h and ℓ, can be the basis also for an iterative RG treatment. Just as the subgrid stress $\tau_{\ell }$ scaled with energy flux rate ε and integral length L is expected to have universal statistical properties in the turbulent inertial range, so too the inertial drag force $f_{h,\ell }$ scaled with friction velocity $u_{\tau }$ and distance y to the wall can be expected to exhibit universal statistics in the inertial sublayer of wall-bounded turbulence. These are both prime problems for investigation by Wilson-type RG methods.

## Footnotes

1 We do not follow the fashion of referring to Onsager’s result on energy conservation for Hölder exponent h>1/3 as the ‘Onsager conjecture’, and we shall instead refer to it as the ‘Onsager theorem’. Onsager did not state this result in his published 1949 paper [5] as a conjecture, but instead he claimed that ‘it is possible to show that’ and he had, in fact, the outlines of a rigorous proof [6,7]. The idea further suggested by Onsager [5] that energy should be dissipated for some Euler solutions with Hölder exponent h≤1/3 is, however, legitimately referred to as the ‘Onsager conjecture’. For a review of the important recent progress on that latter problem by ‘convex integration’ methods, see [8,9].

2 Rigorously, the only solutions of (2.1) known to exist globally in time in three dimensions are the Leray weak solutions [41] and the momentum balance in the global sense (2.2) seems to have never been proved for such solutions! The difficulty is that weak solutions must be smeared with test functions supported in the interior of the domain Ω and thus such solutions know *a priori* nothing about the boundary. Regularity is lacking to apply existing trace theorems to deduce boundary values for Leray solutions. (We thank Vlad Vicol and Theo Drivas for discussions of this point.) It is possible that the mathematical gap is not purely technical and the global balance (2.2) does not hold for all Leray solutions. The situation could be similar to that for kinetic energy balance, where energy dissipation anomalies might occur at *finite* Re if Leray singularities exist [12].

## Data accessibility

This article has no additional data.
